# Preparation of a disulfide-linked precipitative soluble support for solution-phase synthesis of trimeric oligodeoxyribonucleotide 3´-(2-chlorophenylphosphate) building blocks

**DOI:** 10.3762/bjoc.11.171

**Published:** 2015-09-07

**Authors:** Amit M Jabgunde, Alejandro Gimenez Molina, Pasi Virta, Harri Lönnberg

**Affiliations:** 1 Department of Chemistry, University of Turku, FIN-20014 Turku, Finland; 2 current address: Rega Institute for Medical Research, Minderbroedersstraat 10, KU Leuven, 3000- Leuven, Belgium

**Keywords:** disulfide linker, oligodeoxyribonucleotides, phosphotriester chemistry, precipitation, soluble support

## Abstract

The preparation of a disulfide-tethered precipitative soluble support and its use for solution-phase synthesis of trimeric oligodeoxyribonucleotide 3´-(2-chlorophenylphosphate) building blocks is described. To obtain the building blocks, *N*-acyl protected 2´-deoxy-5´-*O*-(4,4´-dimethoxytrityl)ribonucleosides were phosphorylated with bis(benzotriazol-1-yl) 2-chlorophenyl phosphate. The “outdated” phosphotriester strategy, based on coupling of P^V^ building blocks in conjunction with quantitative precipitation of the oligodeoxyribonucleotide with MeOH is applied. Subsequent release of the resulting phosphate and base-protected oligodeoxyribonucleotide trimer 3’-pTpdC^Bz^pdG^ibu^-5’ as its 3’-(2-chlorophenyl phosphate) was achieved by reductive cleavage of the disulfide bond.

## Introduction

Synthetic nucleic acids have been used to regulate gene expression through different mechanisms of action, such as antisense oligonucleotides [1–2], ribozymes [3], interfering RNAs (siRNA) [4–5] and immunostimulatory CpG [6] based therapeutics. At the same time, the interest in detailed understanding of the factors that govern the interaction of nucleic acids with small molecular entities and other biopolymers has increased. In particular, for NMR spectroscopic studies of such interactions, oligonucleotides are often required in quantities that are inconvenient to prepare by laboratory scale solid-phase synthesis. We have previously reported that short oligonucleotides may be conveniently prepared in hundreds of milligrams scale on a soluble tetrakis-*O*-[4-(1,2,3-triazol-1-yl)methylphenyl]pentaerythritol support that precipitates quantitatively from MeOH [7–10]. For example, the “outdated” phosphotriester strategy [11–14], exploiting 3´-(2-chlorophenyl phosphate) building blocks works well on this support [8]. No oxidation step is needed and the coupling cycle, hence, contains only two steps: 5´-deprotection and coupling. To prepare long sequences, it may, however, be necessary to apply convergent solution-phase coupling of oligomeric 3´-(2-chlorophenyl phosphate) building blocks [15]. For the preparation of such building blocks, a 2-hydroxyethyldisulfanyl functionalized support may in principle be used [16–21]. After completion of the chain assembly on the hydroxy function, the disulfide linkage may be reductively cleaved and the phosphate bound 2-mercaptoethyl group is removed. Accordingly, the oligomer expectedly is released in a fully protected form. We now report on the synthesis of such a soluble support, **3**, and show that it allows efficient coupling by the 1-hydroxybenzotriazole promoted phosphotriester coupling [11,22–23] and efficient purification of the support bound product by precipitation from MeOH after each deprotection and coupling step.

## Results and Discussion

**Scheme 1:**
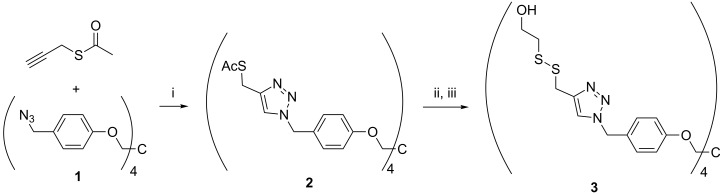
Synthesis of soluble support. Reagents and conditions: (i) CuI, sodium ascorbate, DMAC, 50 °C, 12 h; (ii) butylamine, MeOH, room temperature, 12 h; (iii) 2-(pyridine-2-yldisulfanyl)ethanol, MeCN/DCM/MeOH, 0.5 h.

The synthesis of the disulfide tethered tetrakis-*O*-({4-[(2-hydroxyethyldisulfanyl)methyl]-1*H*-1,2,3-triazol-1-ylmethylphenyl}pentaerythritol support (**3**) is outlined in Scheme 1. Commercially available *S*-propargyl thioacetate was first conjugated to the tetrakis-*O*-[4-(azidomethylphenyl)pentaerythritol support (**1**) [7] by Cu(I) catalyzed 1,3-dipolar cycloaddition [24–25], yielding tetrakis-*O*-({4-[4-[(acetylthiomethyl)]-1*H*-1,2,3-triazol-1-ylmethyl]phenyl}pentaerythritol (**2**) as a thick oil. Careful aminolysis of the thioacetates with butylamine in degassed methanol under inert atmosphere followed by immediate reaction of the exposed mercapto groups with 2-(pyridine-2-yldisulfanyl)ethanol gave the desired tetrapodal soluble support **3**.

**Scheme 2:**
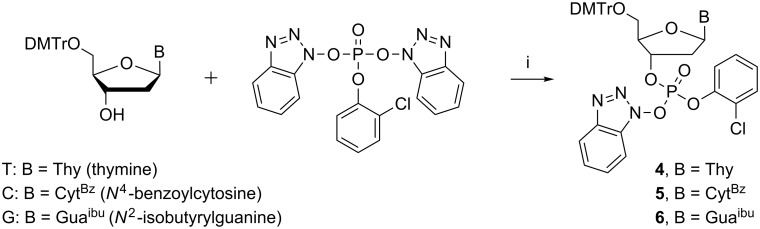
Activation of nucleosides. Reagents and conditions: (i) pyridine, dry dioxane, room temperature, 3 h.

Previously [11] described phosphorylation with bis(benzotriazol-1-yl) 2-chlorophenyl phosphate in 1,4-dioxane was applied to convert commercial thymidine, *N*^4^-benzoyl-2’-deoxycytidine and *N*^2^-isobutyryl-2’-deoxyguanosine into their 3’-(benzotriazol-1-yl 2-chlorophenyl phosphates) (**4**–**6**; Scheme 2). The stock solution of the latter reagent (0.2 mol L^−1^) was prepared as described in our previous report [8].

**Scheme 3:**
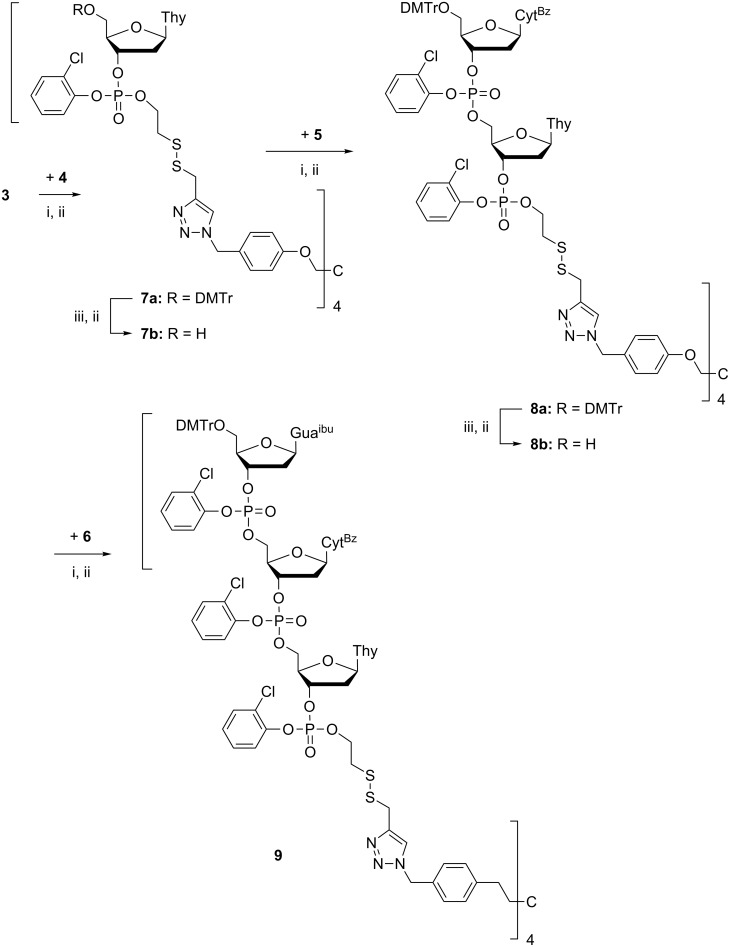
Synthesis of oligonucleotides. Reagents and conditions: (i) **4**–**6**, 1-methylimidazole, dioxane, under N_2_; (ii) precipitation with MeOH; (iii) 1. HCl in MeOH/DCM (1:1); 2. pyridine, solvent evaporation.

**Figure 1:**
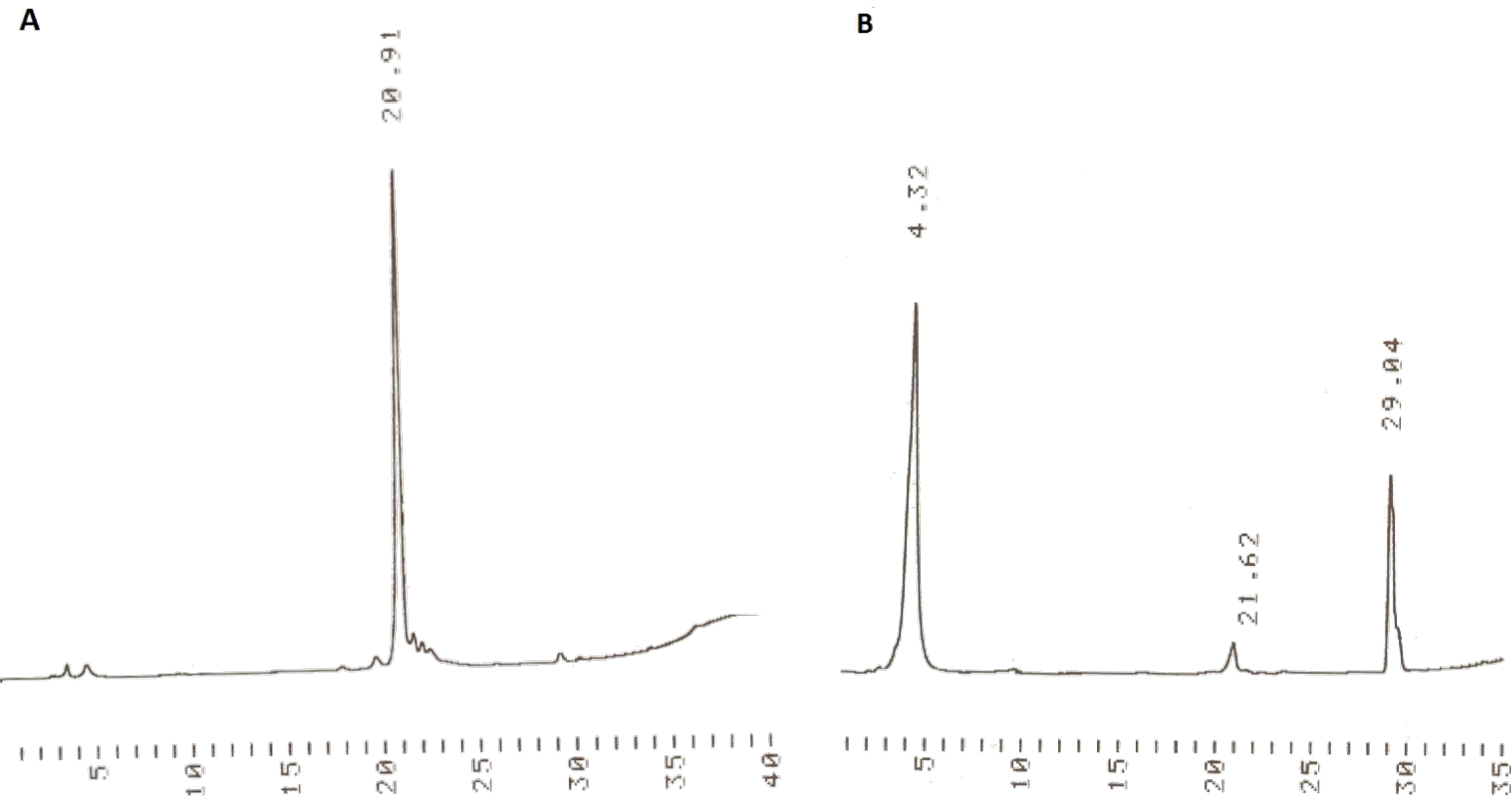
(A) HPLC traces for the precipitated support bearing phosphate-protected 3’-T-5’ monomer having the 5’-hydroxy group detritylated (**7b**), and (B) HPLC traces of the filtrate after precipitation. For the chromatographic conditions, see gradient A in the Experimental section.

A trimeric oligodeoxyribonucleotide containing three different 2’-deoxyribonucleosides was assembled on support **3** as depicted in Scheme 3. The couplings were carried out essentially as described previously [8]. Accordingly, the dried support was treated under nitrogen with the thymidine derived building block **4** (2 equiv per support-bound OH), in dioxane in the presence of 1-methylimidazole. The coupling was completed in 12 h, and the excess of **4** and the coupling reagents were removed by precipitating the support with MeOH to obtain **7a**. According to TLC analysis, the precipitation was quantitative. Detritylation of **7a** with HCl in a 1:1 mixture of MeOH and DCM, followed by neutralization with pyridine, concentration to oil and precipitation from MeOH, afforded **7b**. HPLC-analysis verified the completeness of precipitation (Figure 1).

**Figure 2:**
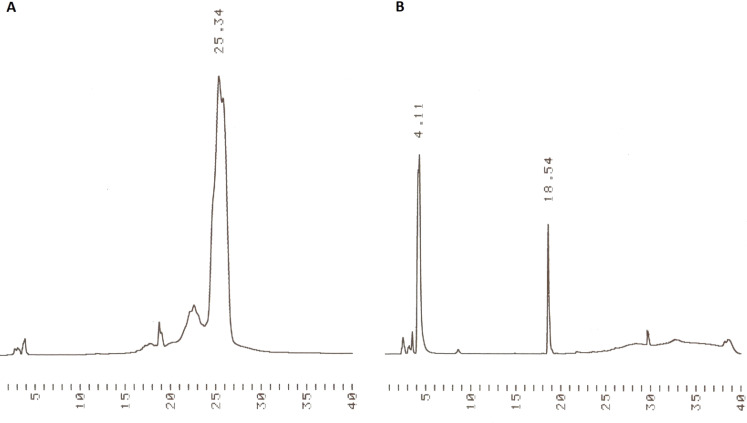
(A) HPLC traces for the precipitated support bearing phosphate-protected 3’-pTpdC^Bz^-5’-*O*-DMTr (**8a**) and (B) HPLC traces of the filtrate after precipitation. For the chromatographic conditions, see gradient B in the Experimental section.

**Figure 3:**
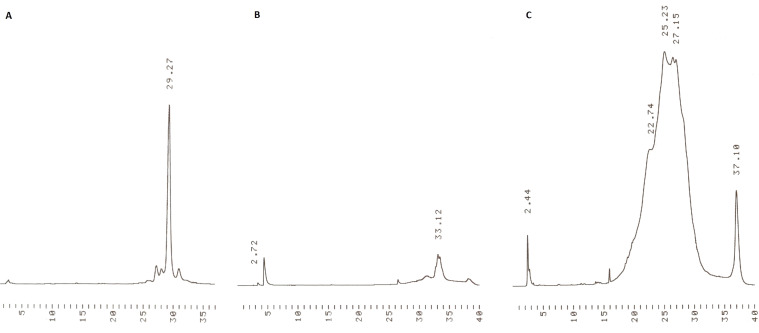
(A) HPLC traces for the precipitated support bearing phosphate-protected 3’-pTpdC^Bz^-5’-OH dimer **8b**, and (B) HPLC traces of the filtrate after precipitation of **8b**. (C) HPLC traces for the precipitated support bearing phosphate-protected 3’-pTpdC^Bz^pdG^ibu^-5’-*O*-DMTr (**9**). For the chromatographic conditions, see gradient A (for **8b** and its filtrate) and gradient B (for **9**) in the Experimental section.

Building blocks **5** and **6** were then coupled similarly to obtain **9** (Scheme 3). The identity and homogeneity of the product were verified by ESIMS and HPLC after each coupling and 5´-deprotection step. The MS data are given in Table 1. Figure 2 and Figure 3 show as an illustrative example the HPLC traces for **8a**, **8b** and **9** precipitated from MeOH and the filtrate of precipitation. As seen, the precipitation is virtually quantitative.

**Table 1:** ESIMS characterization of the support-bound nucleotides indicated in Scheme 3.

Compound	Calculated mass	Observed mass

**7a**	2060.0 [(M − 2H)/2]^2−^	2059.9 [(M − 2H)/2]^2−^
**7b**	1455.3 [(M − 2H)/2]^2−^	1455.2 [(M − 2H)/2]^2−^
**8a**	3067.7 [(M − 2H)/2]^2−^	3067.7 [(M − 2H)/2]^2−^
**8b**	2462.9 [(M − 2H)/2]^2−^	2462.7 [(M − 2H)/2]^2−^
**9**	2724.6 [(M − 3H)/3]^3−^	2724.5 [(M − 3H)/3]^3−^

**Scheme 4:**
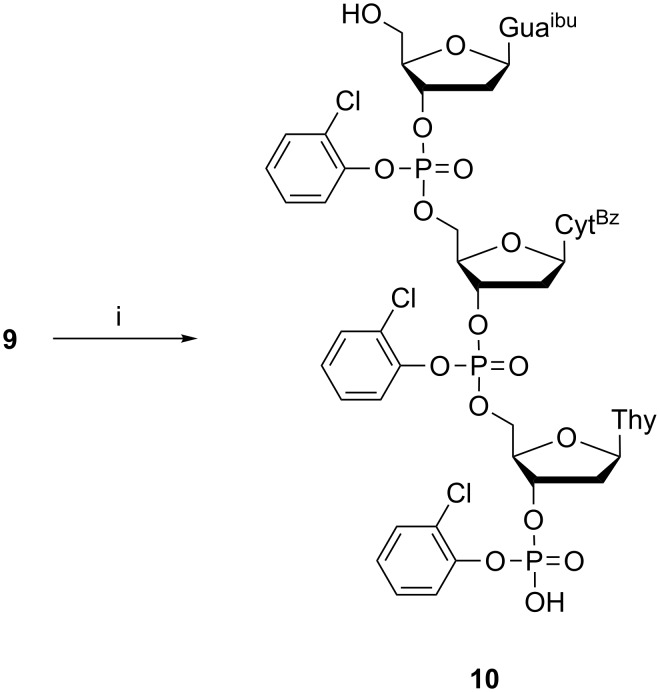
Cleavage of oligonucleotides from support. Reagents and conditions: (i) tris(2-carboxyethyl)phosphine hydrochloride, Et_3_N, MeOH.

The trimer prepared was then released from the support by cleaving the disulfide linkage by TCEP reduction. The phosphate bound 2-mercaptoethyl group was removed spontaneously giving the oligonucleotide trimer expectedly as a in fully protected 3´-(2-chlorophenyl phosphate). While the phosphate and base moiety protections remained intact, the 5´-*O*-(4,4´-dimethoxytrityl) group was unfortunately lost (Scheme 4). It has been previously shown [26] that the disulfide linkage may be cleaved by reduction with dithiotreitol or TCEP at pH buffered to 7.6 without detritylation taking place. Elongated treatment with TCEP appeared, however, to be too harsh. Evidently, the 5´-terminal nucleoside should be inserted as a more acid tolerant 5´-*O*-(4-methoxytrityl) protected building block. The concentrated reaction mixture was washed with MeCN and collected washings having crude trimer were dried and purified by semi-preparative HPLC to afford pure trimer oligodeoxyribonucleotide (**10**).

## Conclusion

Tetrakis-*O*-({4-[4-[(2-hydroxyethyldisulfanylmethyl)]-1*H*-1,2,3-triazol-1-ylmethyl]phenyl}pentaerythritol (**3**) has been synthesized and used as a tetrapodal soluble support for the synthesis of a fully protected trimeric oligodeoxyribonucleotide as a 3´-(2-chlorophenyl phosphate) by the 1-hydroxybenzotriazole promoted phosphotriester chemistry. The support enabled efficient purification of the support-bound intermediates and product by virtually quantitative precipitation from MeOH after each coupling and 5´-*O*-deprotection. However, on assembling the trimeric 3´-(2-chlorophenyl phosphate) building blocks, the 5´-terminal nucleoside should be introduced in a 5´-*O*-(4-methoxytrityl) protected form to prevent detritylation during the reductive cleavage of the trimer from the support.

## Experimental

General: NMR spectra were recorded on a Bruker Avance spectrometer (500 or 400 MHz) at 25 °C. Chemical shifts are given in ppm and referenced relative to the residual solvent signals. RP HPLC conditions: (A) gradient elution from 25% MeCN in 0.1 mol L^−1^ Et_3_NHOAc to 70% MeCN in 0.1 mol L^−1^ Et_3_NHOAc in 25 min, then continued with 70% MeCN in 0.1 mol L^−1^ Et_3_NHOAc to 100% MeCN in 35 min; (B) gradient elution from 25% MeCN in 0.1 mol L^−1^ Et_3_NHOAc to 100% MeCN in 25 min, then continued with MeCN; An analytical C-18 RP column (250 × 4.6 mm, 5 μm; flow rate 1.0 mL min^−1^; λ = 260 nm) was used. Reactions were monitored by TLC (Merck, silica gel 60 F_254_), using short wavelength UV or charring with 10% aq H_2_SO_4_ for detection (system A: 5% MeOH in CH_2_Cl_2_; system B: 10% MeOH in CH_2_Cl_2_). Mass spectra were recorded with a Bruker Daltonics MicrOTOFQ spectrometer using ESI mode.

**Tetrakis-*O*-(4-azidomethylphenyl)pentaerythritol (1)** was prepared as described previously [7].

**Tetrakis-*O*-{4-[4-(acetylthiomethyl)-1*H*-1,2,3-triazol-1-ylmethyl]phenyl}pentaerythritol (2): ***S*-Propargyl thioacetate (1.73 mL, 15.0 mmol) was added to the solution of compound **1** (1.00 g, 1.51 mmol), sodium ascorbate (30 mg, 0.15 mmol) and CuI (115 mg, 0.605 mmol) in dry DMAc (5.0 mL) in a Pyrex tube. The tube was degassed via three freeze–pump–thaw cycles, and the mixture was stirred at 50 °C for 12 h. Water (10 mL) was added to the reaction mixture and extracted with ethyl acetate (20 mL × 3). The combined organic layer was washed with saturated NaHCO_3_, dried with Na_2_SO_4_, and the solvents were evaporated to dryness. The residue was purified by silica gel chromatography (CH_2_Cl_2_/MeOH, 97:3 v/v) to give **2** (1.2 g, 70%) as a white foam. ^1^H NMR (400 MHz, CDCl_3_) δ 7.35 (s, 4H, H5 of triazole), 7.18 (d, *J* = 8.6 Hz, 8H, H2&H6 of Ph), 6.89 (d, *J* = 8.6 Hz, 8H, H3&H5 of Ph), 5.38 (s, 8H, N-C*H*_2_-Ph), 4.32 (s, 8H, AcS-C*H*_2_-), 4.13 (s, 8H, C*H*_2_-pentaerythritol), 2.31 (s, 12H, -SAc); ^13^C NMR (125 MHz, CDCl_3_) δ 195.2, 158.9, 144.6, 129.7, 127.2, 122.1, 115.1, 66.4, 53.6, 44.7, 30.4, 23.9 ppm; ESIMS *m*/*z*: [M + H]^+^ calcd for C_53_H_57_N_12_O_8_S_4_, 1117.33, found, 1117.36; *m*/*z*: [M + Na]^+^ calcd for C_53_H_56_N_12_NaO_8_S_4_, 1139.31; found, 1139.32.

**Tetrakis-*O*-{4-[4-(2-hydroxyethyldisulfanylmethyl)-1*H*-1,2,3-triazol-1-ylmethyl]phenyl}pentaerythritol (3)**. Degassed butylamine in MeOH (0.21 mL, 1.0 mol L^−1^, 2.15 mmol) was added to the stirred solution of compound **2** (600 mg, 0.537 mmol) in degassed MeOH. The mixture was stirred under N_2_ for 12 h. Degassed MeCN/DCM (5 mL, 1:1 v/v) was added and the mixture was neutralized with dry acidic ion exchange resin and filtered off. The solvent was removed by evaporation and the crude product was used in the next reaction without purification. 2-(Pyridine-2-yldisulfanyl)ethanol (581 mg, 3.11 mmol) was added to the crude compound in degassed MeCN/DCM/MeOH (4 mL, 2:1:1 v/v/v) under N_2_. The mixture was stirred for 30 min and the progress was monitored by TLC. Solvents were removed by evaporation and the product was purified by silica gel chromatography (DCM/MeOH, 95:5 v/v) to give **3** (120 mg, 19%) as a thick oil. ^1^H NMR (500 MHz, CDCl_3_) δ 7.40 (s, 4H, H5 of triazole), 7.18 (d, *J* = 8.6 Hz, 8H, H2&H6 of Ph), 6.88 (d, *J* = 8.6 Hz, 8H, H3&H5 of Ph), 5.42 (s, 8H, N-C*H*_2_-Ph), 4.30 (s, 8H, S-C*H*_2_-triazole), 3.97 (s, 8H, C*H*_2_-pentaerythritol), 3.79 (t, *J* = 5.5 Hz, 8H, C*H*_2_OH), 2.73 (br t, 8H, S-C*H*_2_CH_2_) ppm; ^13^C NMR (100 MHz, CDCl_3_) δ 158.9, 144.2, 129.6, 127.2, 121.9, 115.2, 66.4, 59.9, 53.7, 44.8, 42.5, 32.8 ppm; ESIMS *m*/*z*: [M + H]^+^ calcd for C_53_H_65_N_12_O_8_S_8_, 1253.28; found, 1253.27; *m*/*z*: [M + Na]^+^ calcd for C_53_H_64_N_12_NaO_8_S_8_, 1275.26; found, 1275.26.

**Bis(benzotriazol-1-yl) 2-chlorophenyl phosphate:** The couplings were carried out essentially as described previously [8]. A solution of 2-chlorophenyl phosphorodichloridate (7.6 mmol, 1.88 g) in anhydrous dioxane (5.75 mL) was added in one portion to a suspension of 1-hydroxybenzotriazole (15.2 mmol, 2.06 g; dried in vacuo over P_2_O_5_ at 55 °C for 3 d) and pyridine (15 mmol, 1.2 mL) in anhydrous dioxane (30 mL). The reaction mixture was stirred for 2 h, and the precipitate was filtered off under anhydrous conditions to give a stock solution of bis(benzotriazol-1-yl) 2-chlorophenyl phosphate (0.2 mol L^−1^) as a clear colorless liquid. The solution (*ρ* = 1.057 g L^−1^) could be stored for several weeks at −20 °C.

**General procedure for the coupling of 1-hydroxybenzotriazole-activated phosphotriester building blocks:** The coupling cycle was analogous to that described previously [8]. 5’-*O*-(4,4’-Dimethoxytrityl)thymidine (0.30 g, 0.54 mmol) was dried by co-evaporation with anhydrous pyridine (3 × 5 mL) and concentrated to a small volume followed by the addition of the stock solution of bis(benzotriazol-1-yl) 2-chlorophenyl phosphate in dioxane (0.54 mmol, 0.2 mol L^−1^, 2.72 mL), giving 5’-*O*-(4,4’-dimethoxytrityl)thymidine 3’-(benzotrizol-1-yl 2-chlorophenyl phosphate) **4** in dioxane. The formation of a product with zero mobility on TLC (system B) indicated that the reaction was complete. In a separate vessel, support **3** (0.068 mmol, 0.086 g) was dried by co-evaporation with anhydrous pyridine (3 × 5 mL), and then **4** in dioxane and 1-methylimidazole (2 mmol, 0.218 mL) were added under nitrogen. The reaction mixture was stirred for 2 h to obtain the tetravalent nucleoside cluster **7a**, transferred to a stoppered 50 mL plastic tube, and MeOH (46 mL) was added. The precipitate formed was kept at −20 °C overnight, isolated by centrifugation, and dried to give **7a** (0.20 g, 70%) as a white solid. The precipitate and supernatant were analyzed by HPLC to verify the completeness of precipitation and the identity of the precipitate was verified by ESIMS (Table 1).

**General procedure for detritylation:** The detritylation cycle was analogous to that described previously [8].Tetravalent **s**upport-bound thymidine monomer **7a** (0.048 mmol, 0.20 g) was dissolved in a mixture of DCM and MeOH (1:1 v/v, 25 mL), and HCl in MeOH (0.115 mL of 1.25 mol L^−1^ solution) was added portion wise. The reaction was monitored by TLC (system A). Once completed, the reaction mixture was neutralized with pyridine (1 mL), and the liquid was concentrated. The resulting oil was dissolved in DCM/MeOH (1:1, 3 mL), and MeOH was added (40 mL). The precipitate formed was kept at –20 °C overnight, collected by centrifugation and dried to give the product **7b** (0.120 g, 85%) as a white solid. The precipitate and supernatant were analyzed by HPLC (Figure 1) and the precipitate by ESIMS (Table 1).

**Cleavage from tetravalent soluble support:** To the solution of support-bound trimer **9** (0.0061 mmol, 0.045 g) in MeOH (1 mL), triethylamine (1.44 mmol, 0.2 mL) and tris(2-carboxyethyl)phosphine (0.027 mmol, 0.008 g), stirred for 3 h, and then volatiles were removed under reduced pressure. The residue was stirred with CH_3_CN (3 mL) for 15 min and the precipitated support was removed by filtration, the filtrate was concentrated and dried in vacuo to give a yellow oil. The oily residue was purified by semi-preparative HPLC to afford the phosphate protected trimer oligodeoxyribonucleotide **10** (20 mg, 57%). ESIMS *m*/*z*: [M + H]^+^ calcd for C_58_H_59_N_10_O_22_P_3_, 1445.21; found, 1445.20; *m*/*z*: [M + Na]^+^ calcd for C_58_H_58_N_10_NaO_22_P_3_, 1467.19; found, 1467.18.

## Supporting Information

File 1 ^1^H NMR, ^13^C NMR and DEPT spectra for **2**, and **3**, ^31^P NMR spectra of **10**, mass spectra for **2, 3, 7a**–**10**.
