# Supplementary material for: Preparation of a disulfide-linked precipitative soluble support for solution-phase synthesis of trimeric oligodeoxyribonucleotide 3´-(2-chlorophenylphosphate) building blocks
Source: Beilstein J Org Chem. 2015 Sep 7;11:1553–60. doi: 10.3762/bjoc.11.171 (PMC4660909; doi:10.3762/bjoc.11.171)
Supplement: File 1 — 1H NMR, 13C NMR and DEPT spectra for 2, and 3, 31P NMR spectra of 10, mass spectra for 2, 3, 7a–10. [file Beilstein_J_Org_Chem-11-1553-s001.pdf]

Supporting Information

for

Preparation of a disulfide linked precipitative soluble support for solution-phase synthesis of trimeric oligodeoxyribonucleotide 3´-(2-chlorophenylphosphate) building blocks

Amit M. Jabgunde[1,2]*, Alejandro Gimenez Molina[1], Pasi Virta[1] and Harri Lönnberg[1]

Address: [1]Department of Chemistry, University of Turku, FIN-20014 Turku, Finland and [2]current address: Rega Institute for Medical Research, Minderbroedersstraat 10, KU Leuven, 3000- Leuven, Belgium

Email: Amit M. Jabgunde - amit.jabgunde@gmail.com

*Corresponding author

^{1}H NMR, ^{13}C NMR and DEPT spectra for 2, and 3, ^{31}P NMR spectra of 10, mass spectra for 2, 3, 7a–10

Contents

Figure S1. ^{1}H and ^{13}C spectra of **2** S2

Figure S2. DEPT spectra of **2** S3

Figure S3. ^{1}H and ^{13}C spectra of **3** S4

Figure S4. DEPT spectra of **3** S5

Figure S5. ^{31}P spectra of **10** S6

Figure S6. MS (ESI) spectrum of **2** S7

Figure S7. MS (ESI) spectrum of **3** S8

Figure S8. MS (ESI) spectrum of **7A** S9

Figure S9. MS (ESI) spectrum of **7B** S10

Figure S10. MS (ESI) spectrum of **8A** S11

Figure S11. MS (ESI) spectrum of **8B** S12

Figure S12. MS (ESI) spectrum of **9** S13

Figure S13. MS (ESI) spectrum of **10** S14

SpinWorks 3: Compound 2, 1H NMR, CDCl3, 400 MHz

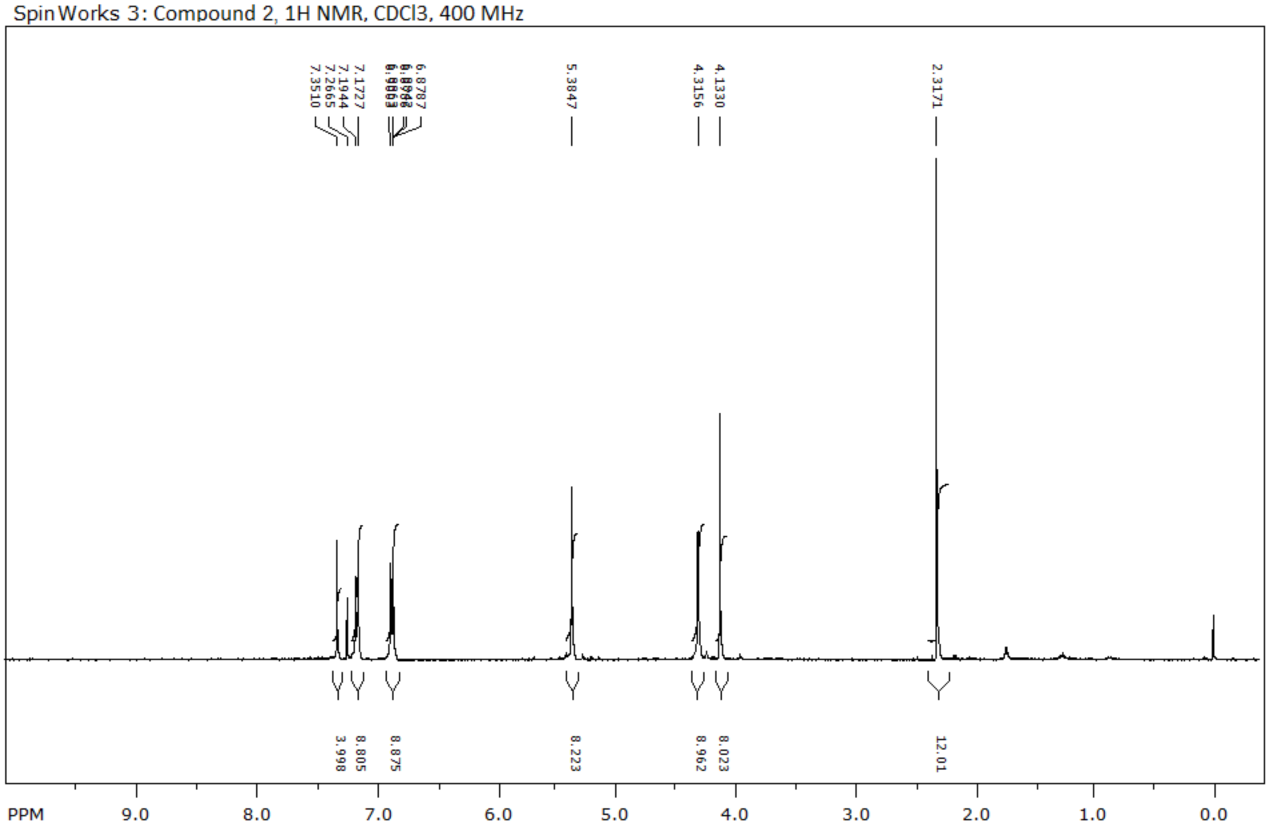

SpinWorks 3: Compound 2, 13C NMR, CDCl3, 125 MHz

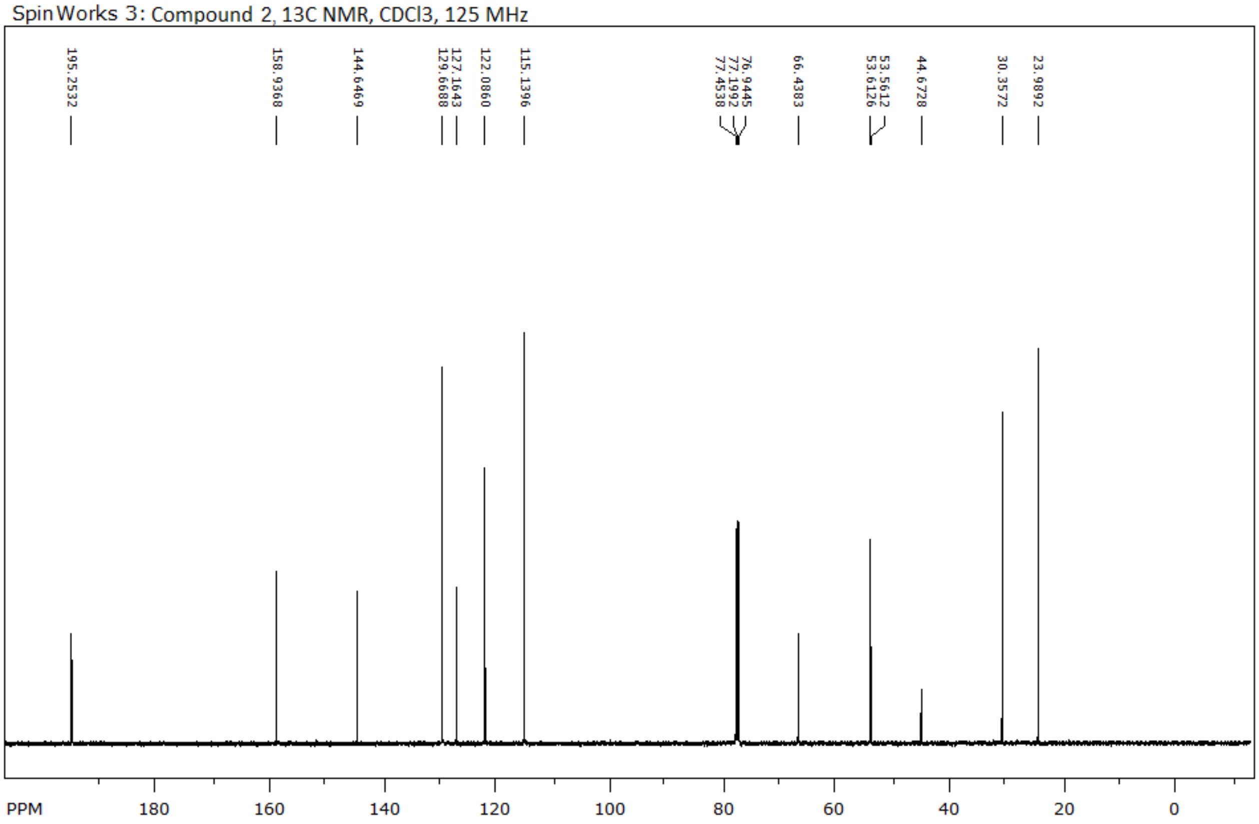

Figure S1: ^{1}H and ^{13}C spectra of **2**.

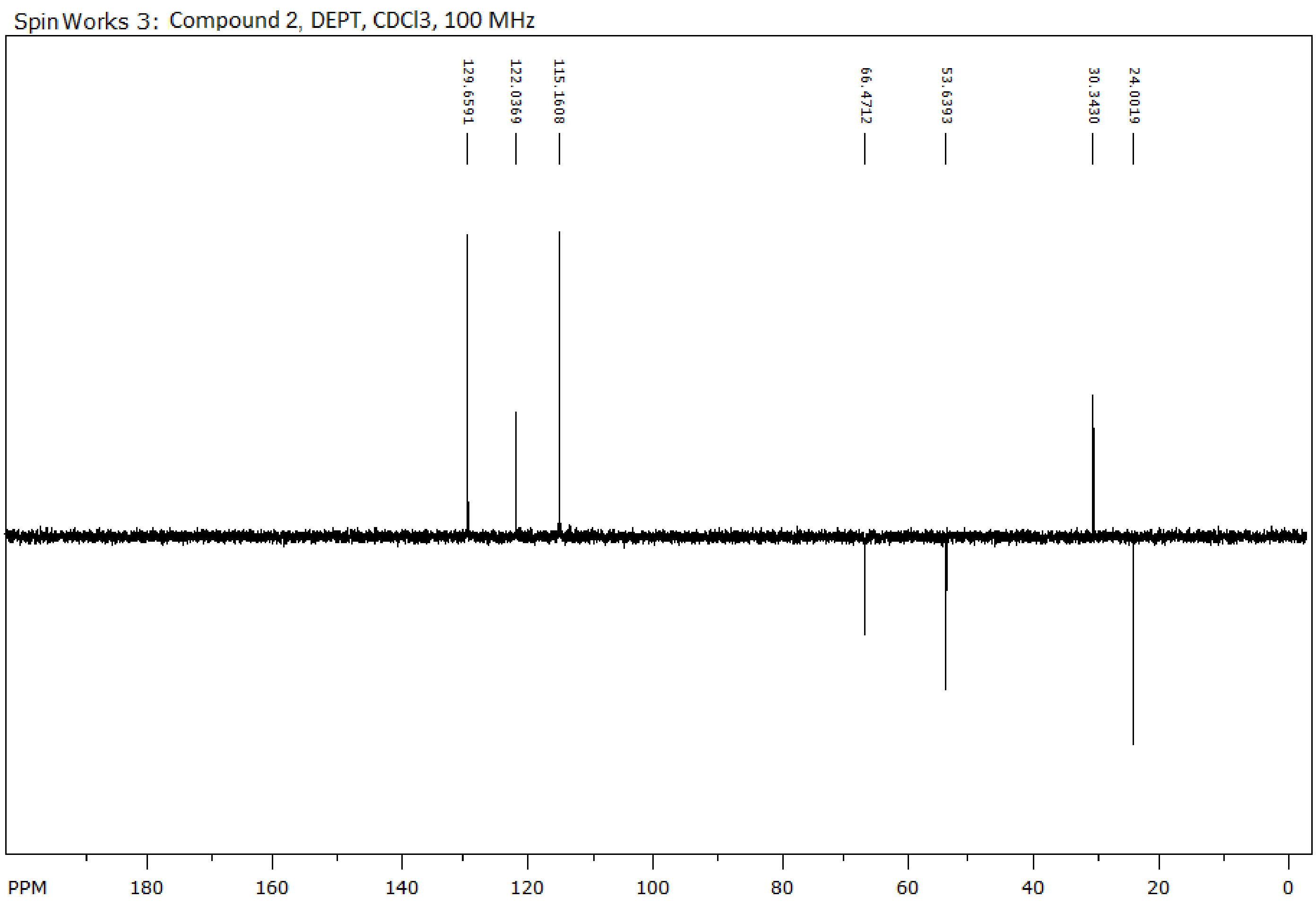

Figure S2: DEPT spectra of **2**.

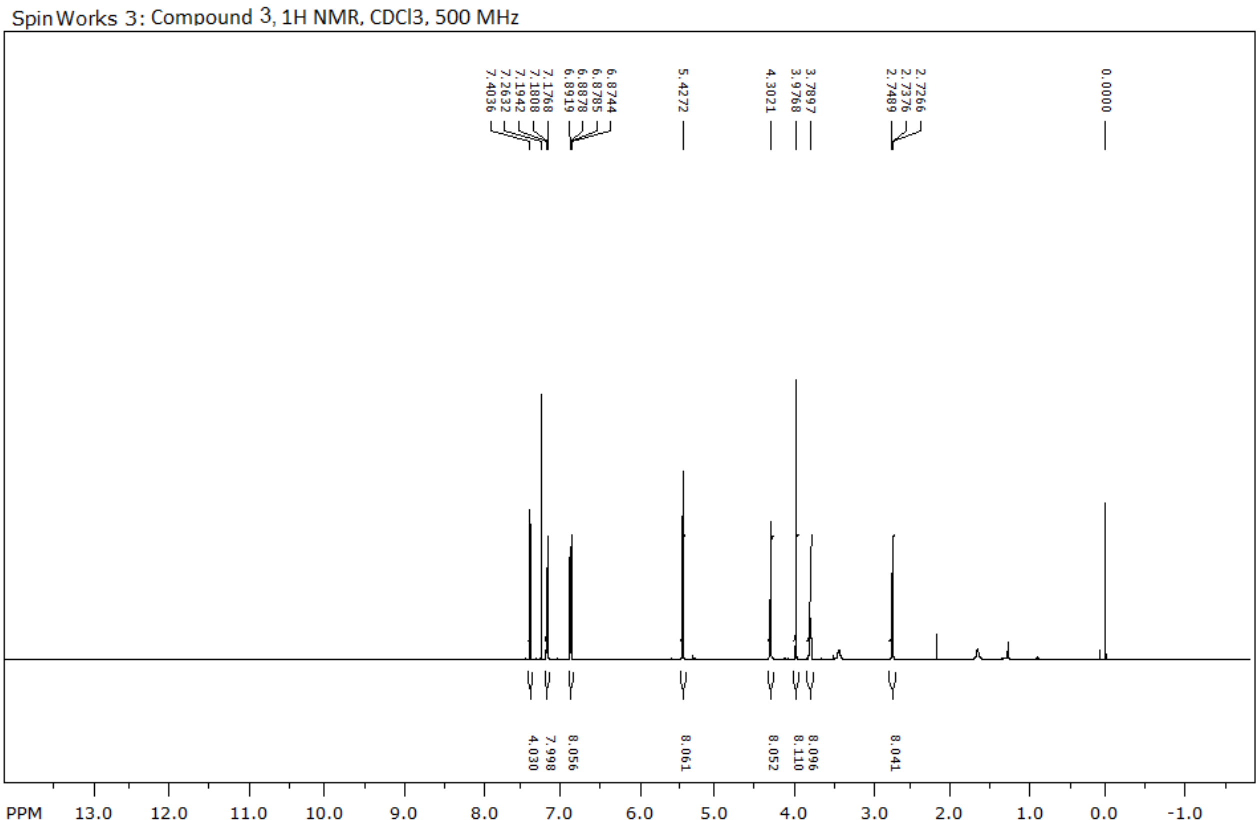

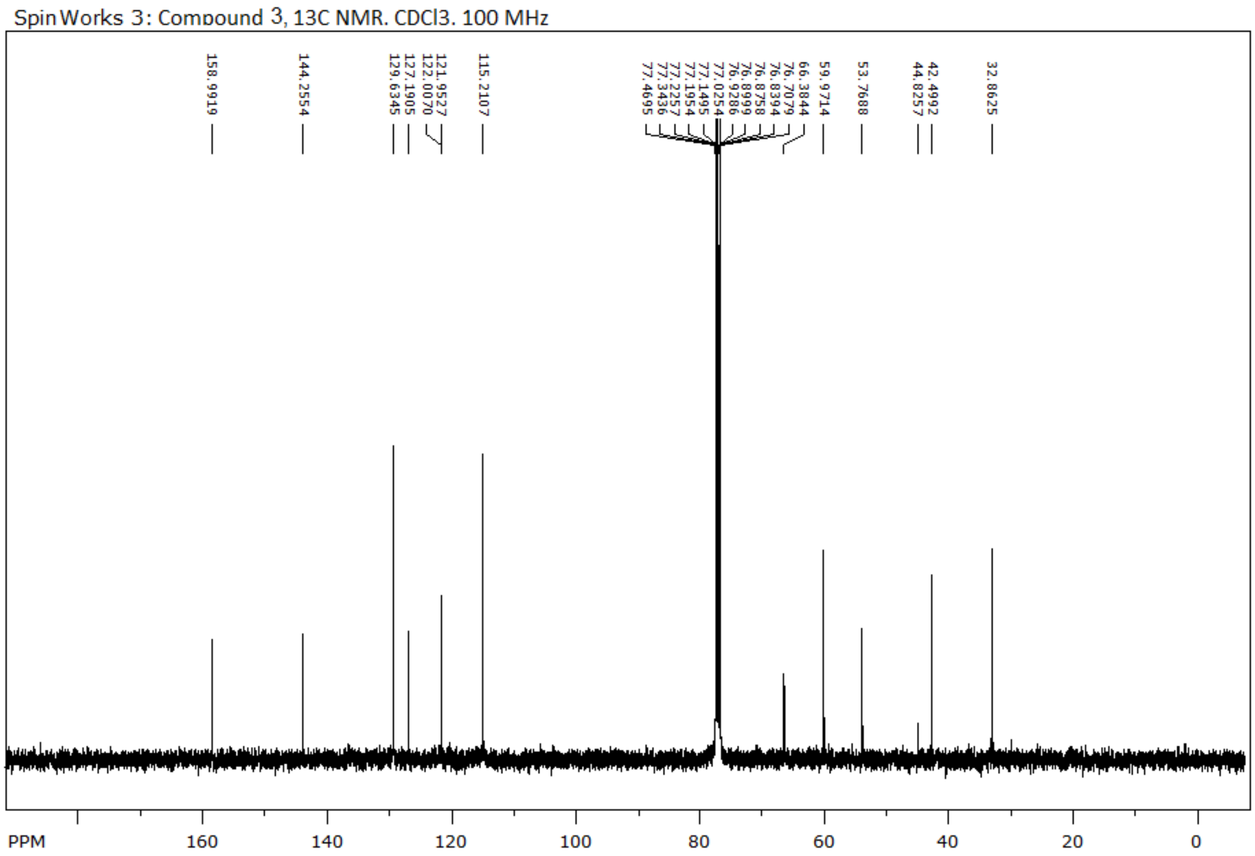

Figure S3: ^{1}H and ^{13}C spectra of **3**.

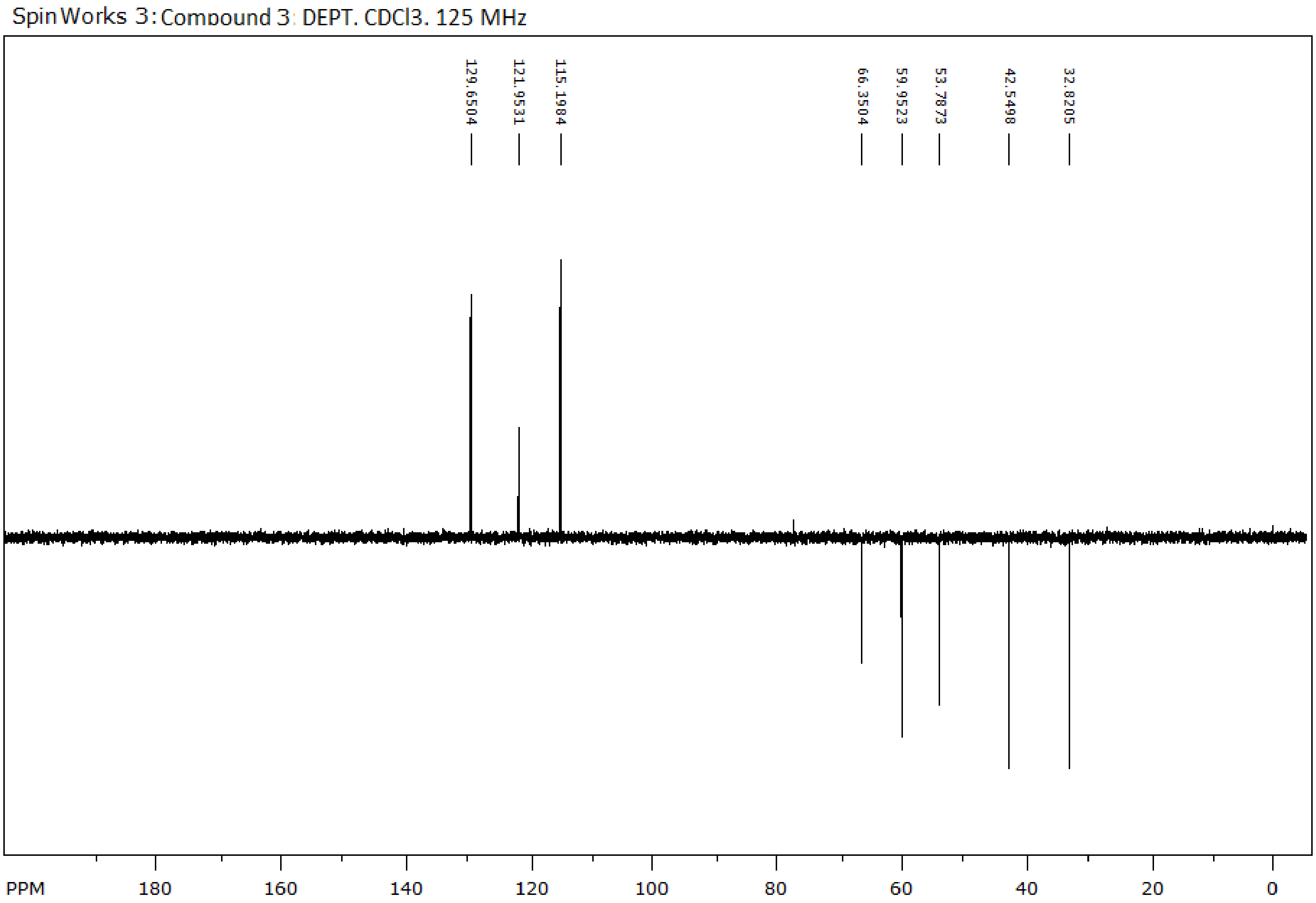

Figure S4: DEPT spectra of **3**.

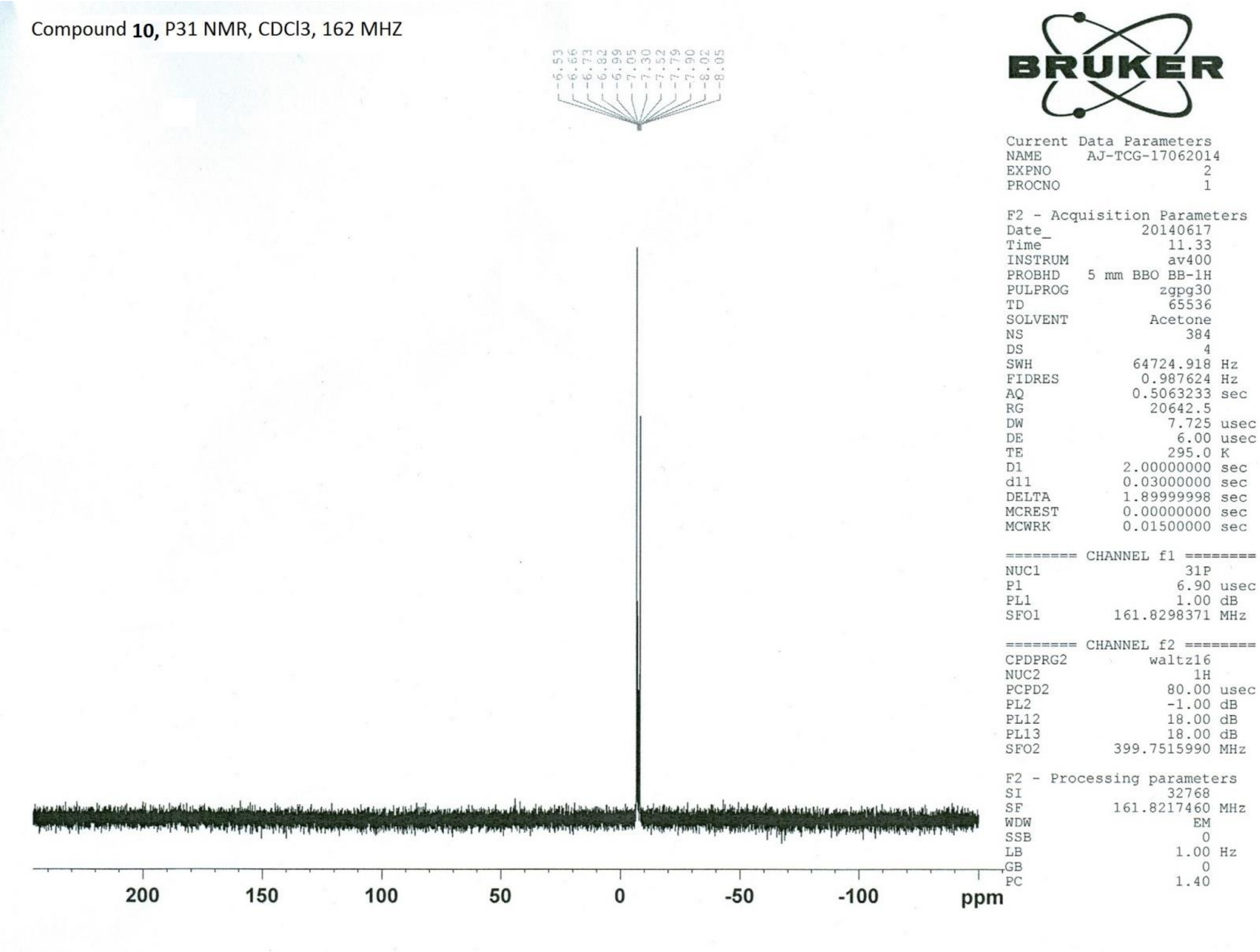

Figure S5: ^{31}P spectra of **10**.

Generic Display Report

Analysis Info

Analysis Name	D:\Data\Amit\Jan-Mar 2013\AJ-Click-Fin- pos19.0313.d	Acquisition Date	3/19/2013 2:41:08 PM
Method	tune_wide pos 12082011working.m	Operator	Bruker07
Sample Name	AJ-Click-Fin- pos19.0313	Instrument	micrOTOF-Q
Comment			

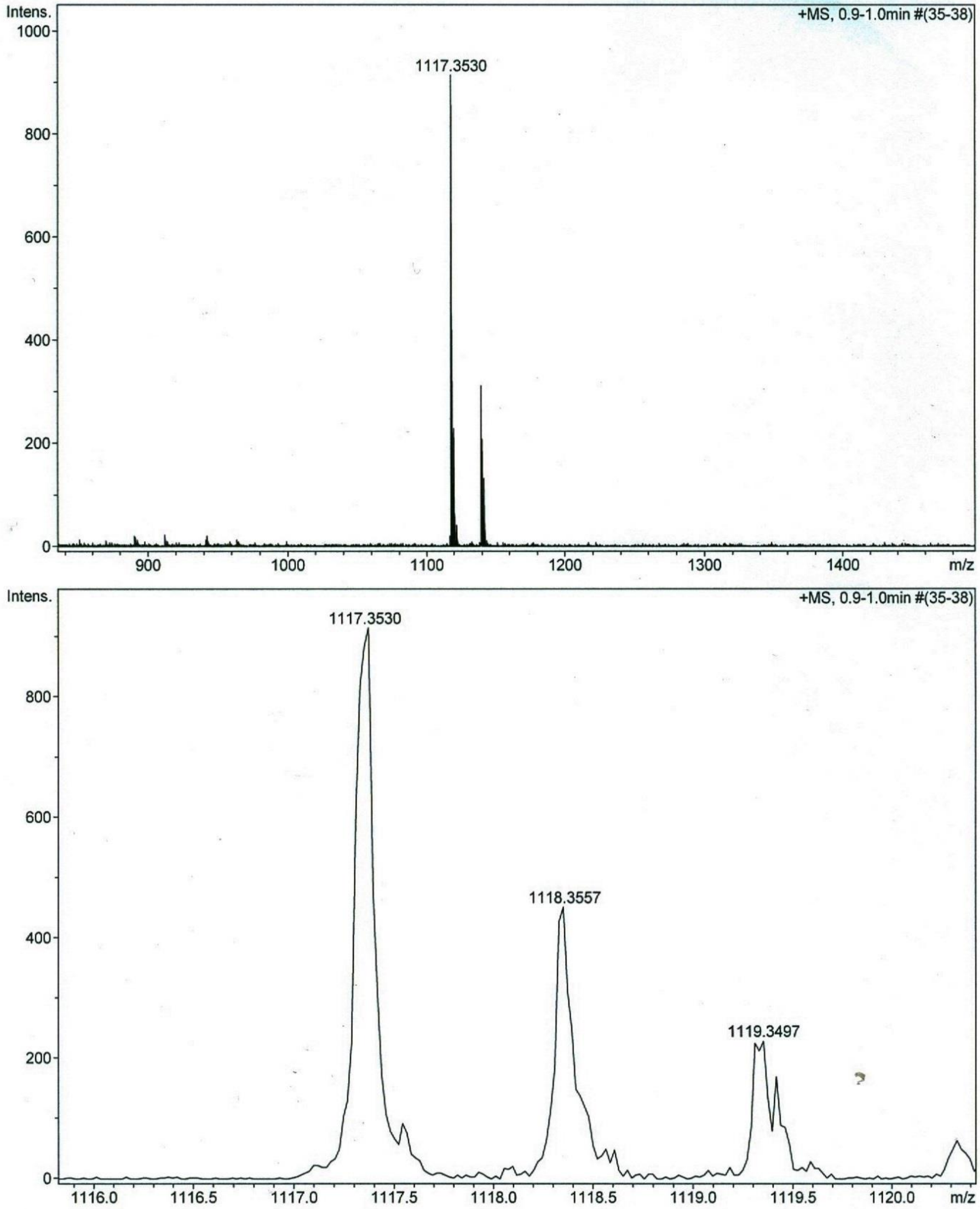

Figure S6: MS (ESI) spectrum of **2**.

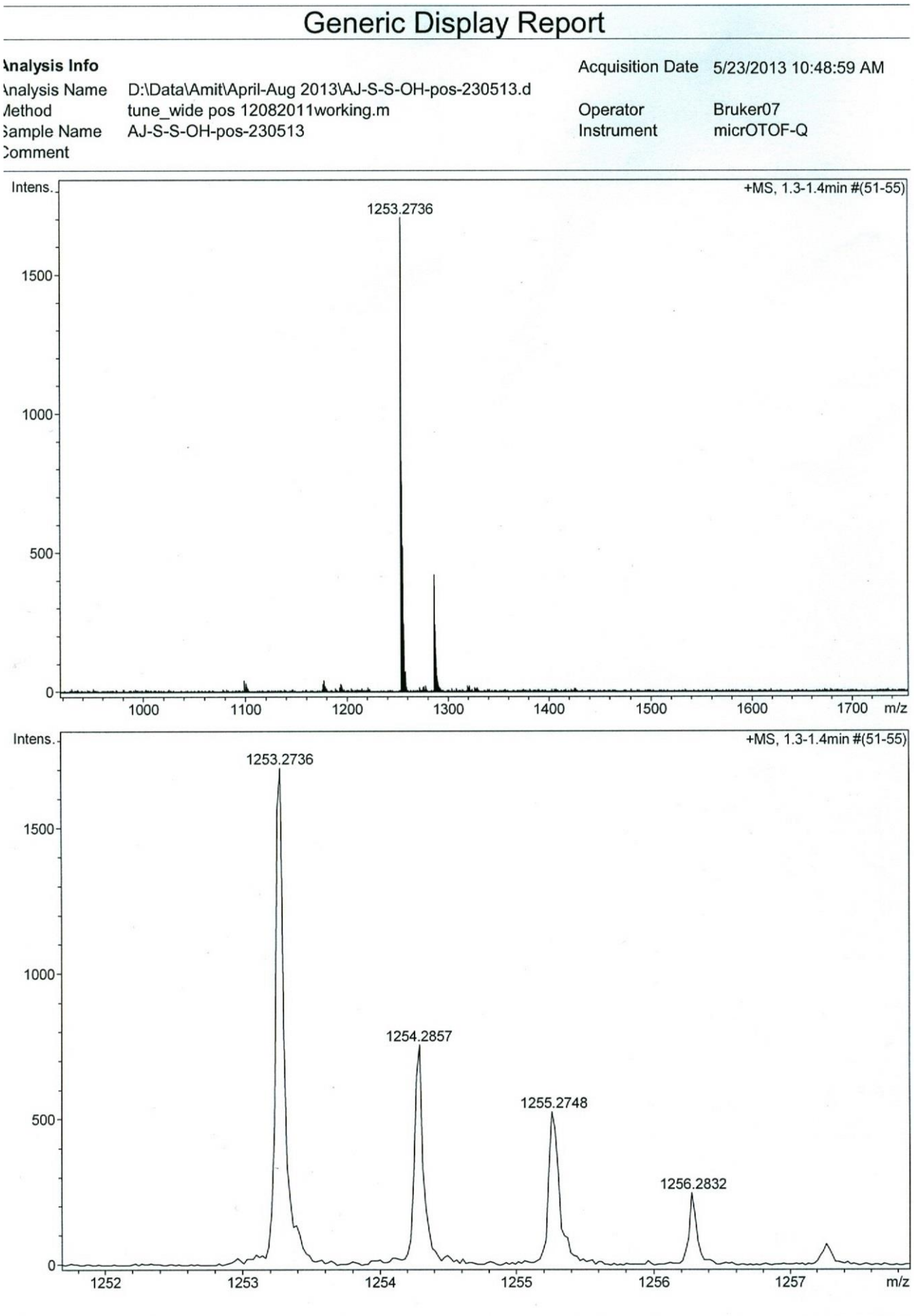

Figure S7: MS (ESI) spectrum of **3**.

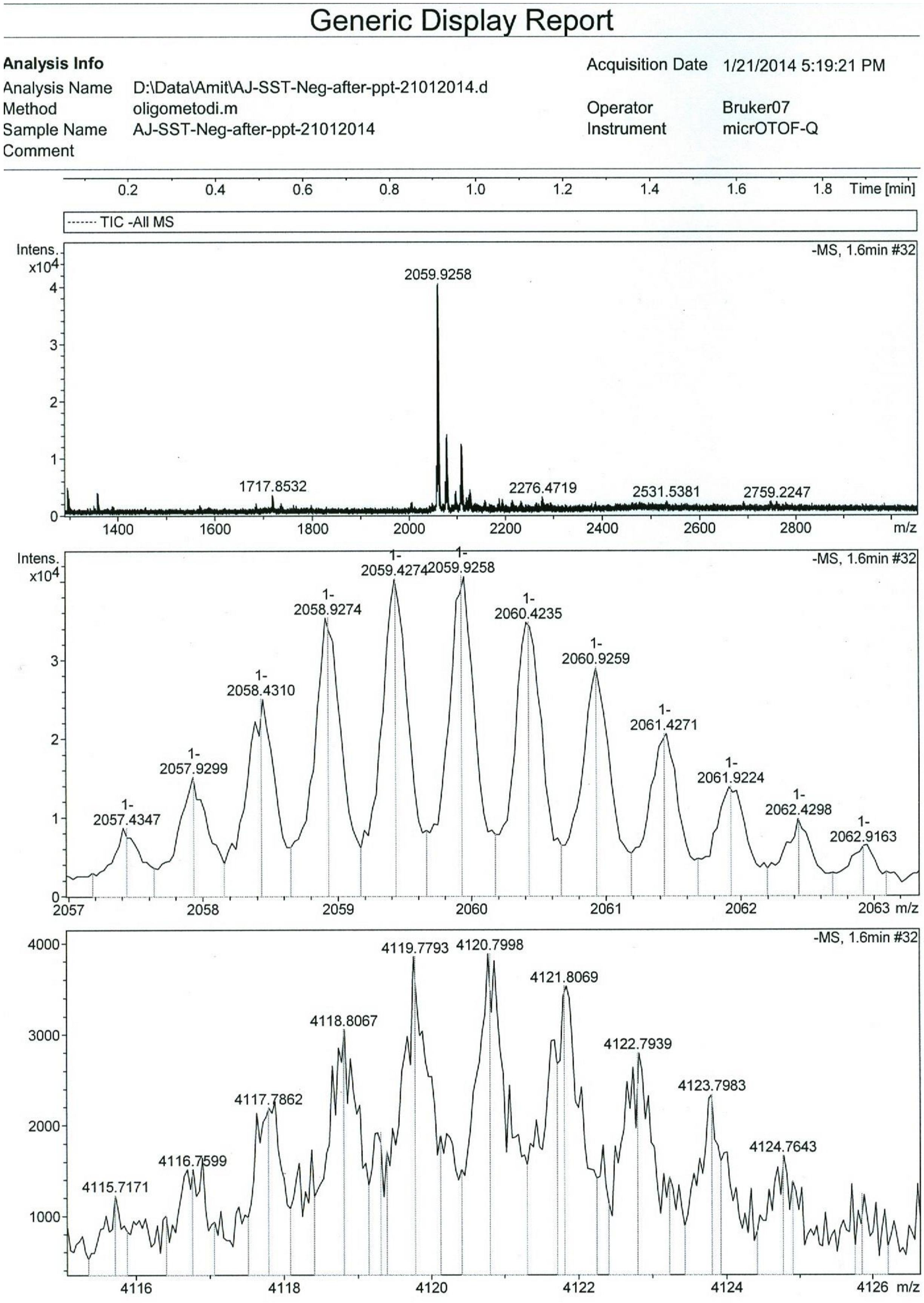

Figure S8: MS (ESI) spectrum of **7A**.

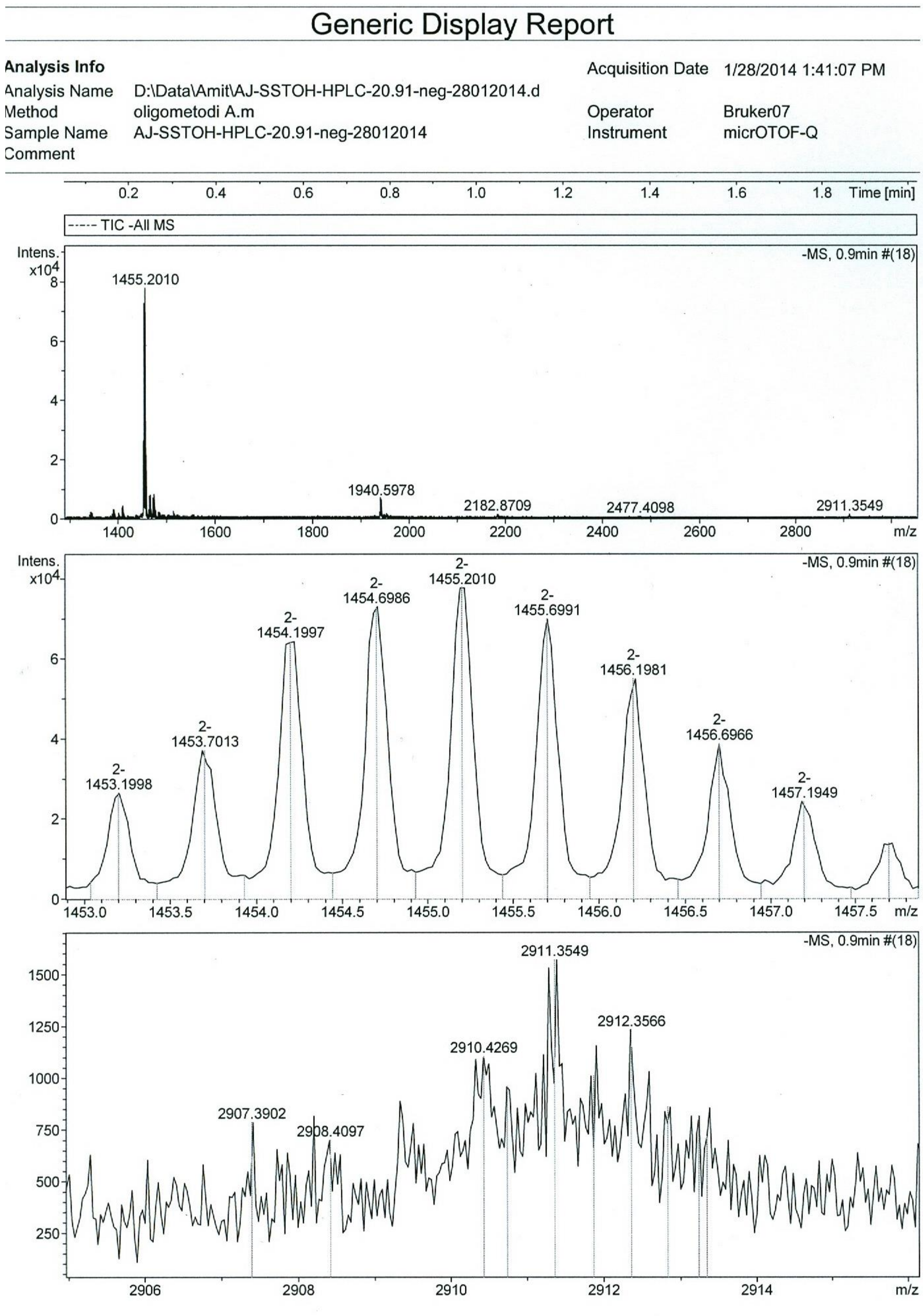

Figure S9: MS (ESI) spectrum of **7B**.

Generic Display Report

Analysis Info		Acquisition Date	2/5/2014 10:49:56 AM
Analysis Name	D:\Data\Amit\AJ-SSTCDMTr-HPLC-31.76-05022014-neg.d		
Method	oligometodi.m	Operator	Bruker07
Sample Name	AJ-SSTCDMTr-HPLC-31.76-05022014-neg	Instrument	micrOTOF-Q
Comment			

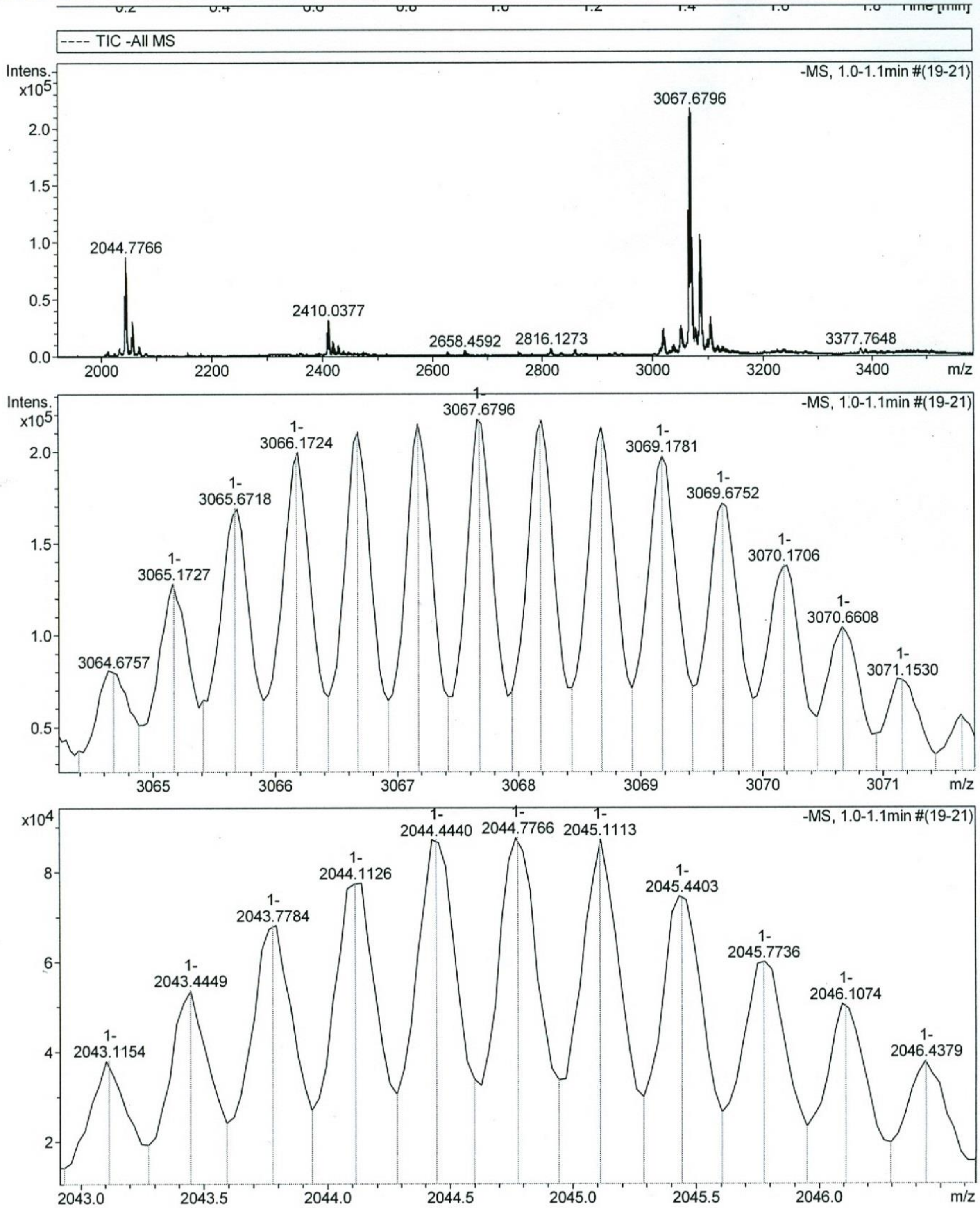

Figure S10: MS (ESI) spectrum of **8A**.

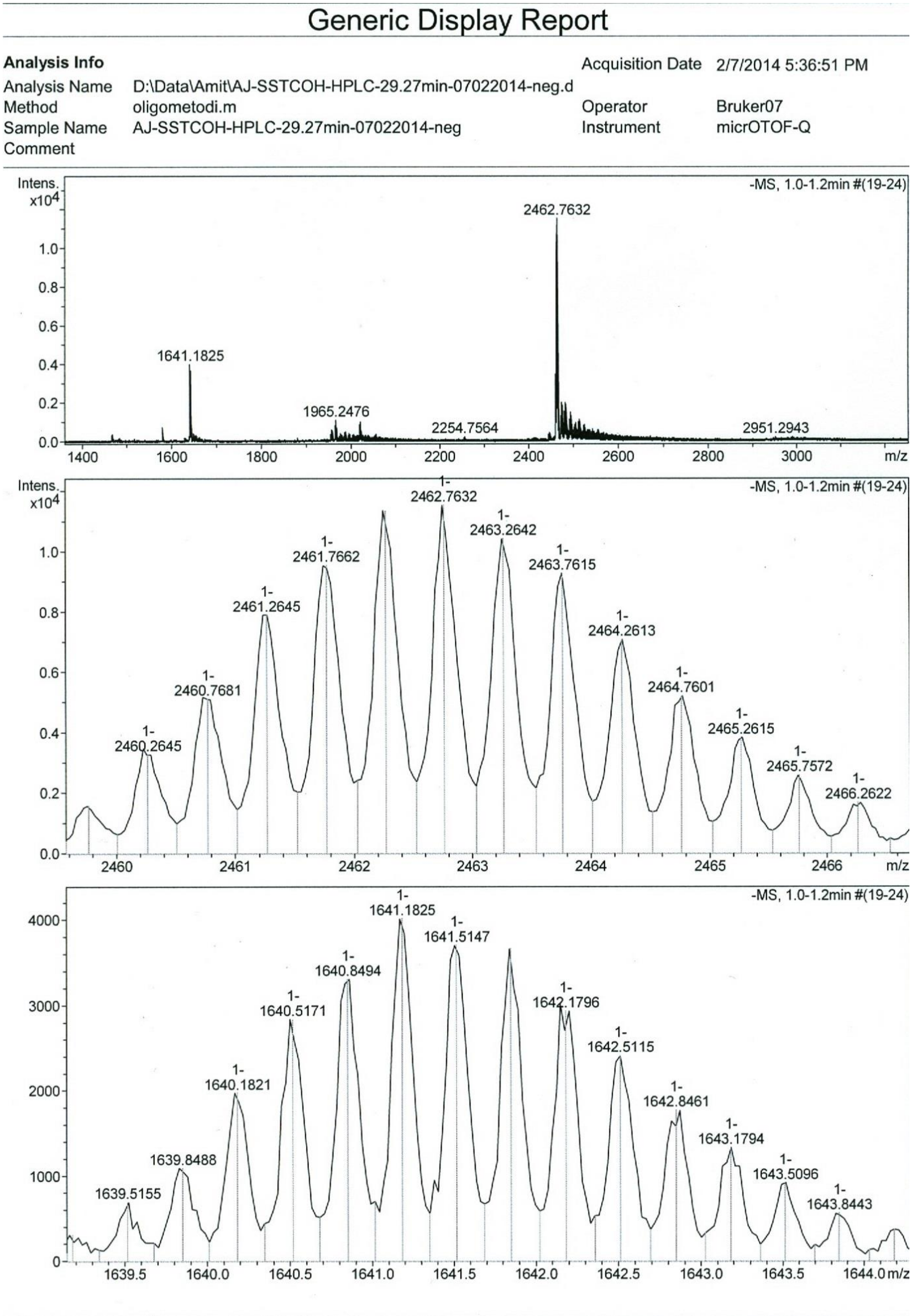

Figure S11: MS (ESI) spectrum of **8B**.

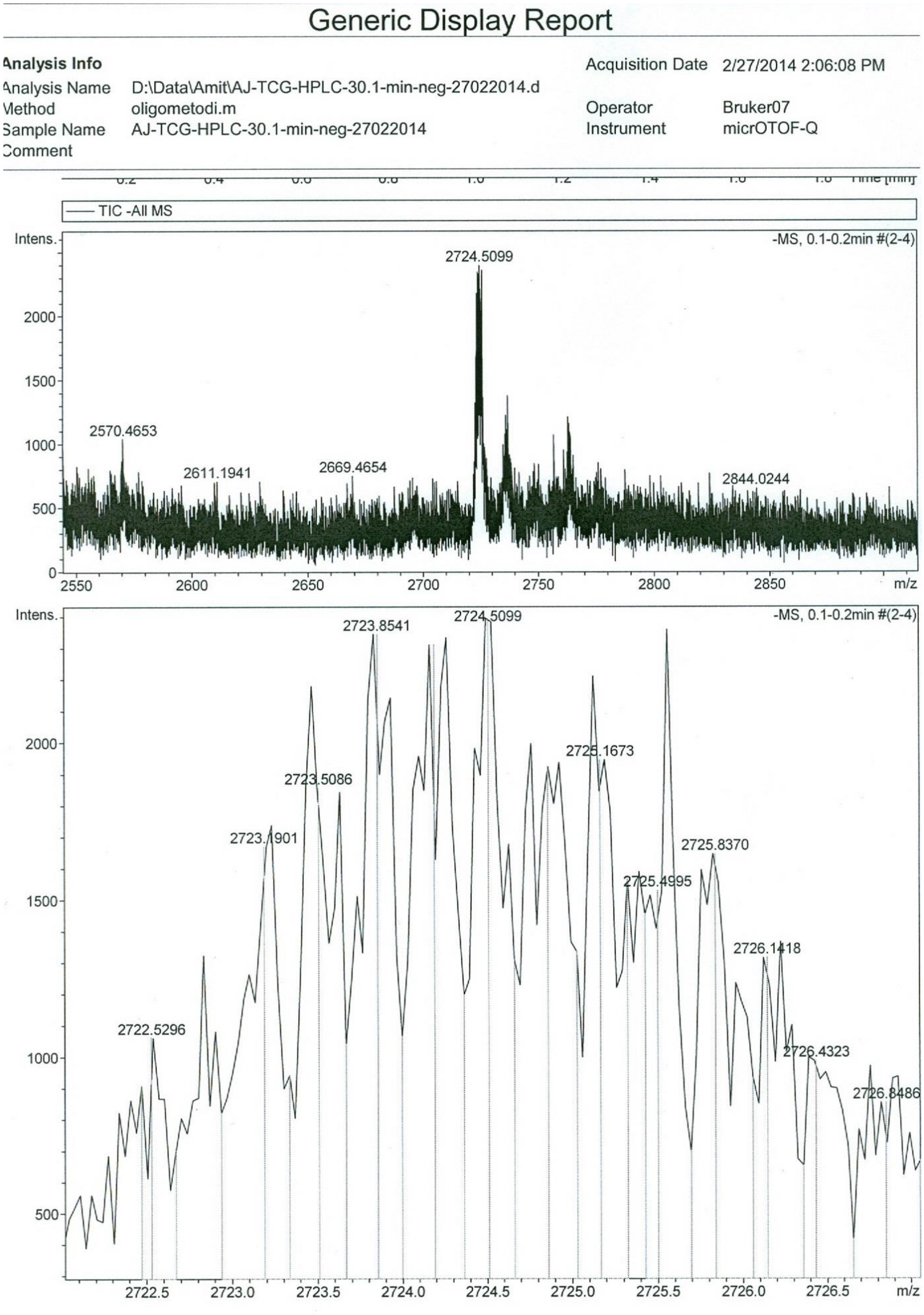

Figure S12: MS (ESI) spectrum of **9**.

Generic Display Report

Analysis Info

Analysis Name	D:\Data\Amit\AJ-TCG-HPLC-16.78.12062014-pos.d	Acquisition Date	6/12/2014 9:39:46 AM
Method	tune_wide pos 12082011working.m	Operator	Bruker07
Sample Name	AJ-TCG-HPLC-16.78.12062014-pos	Instrument	micrOTOF-Q
Comment			

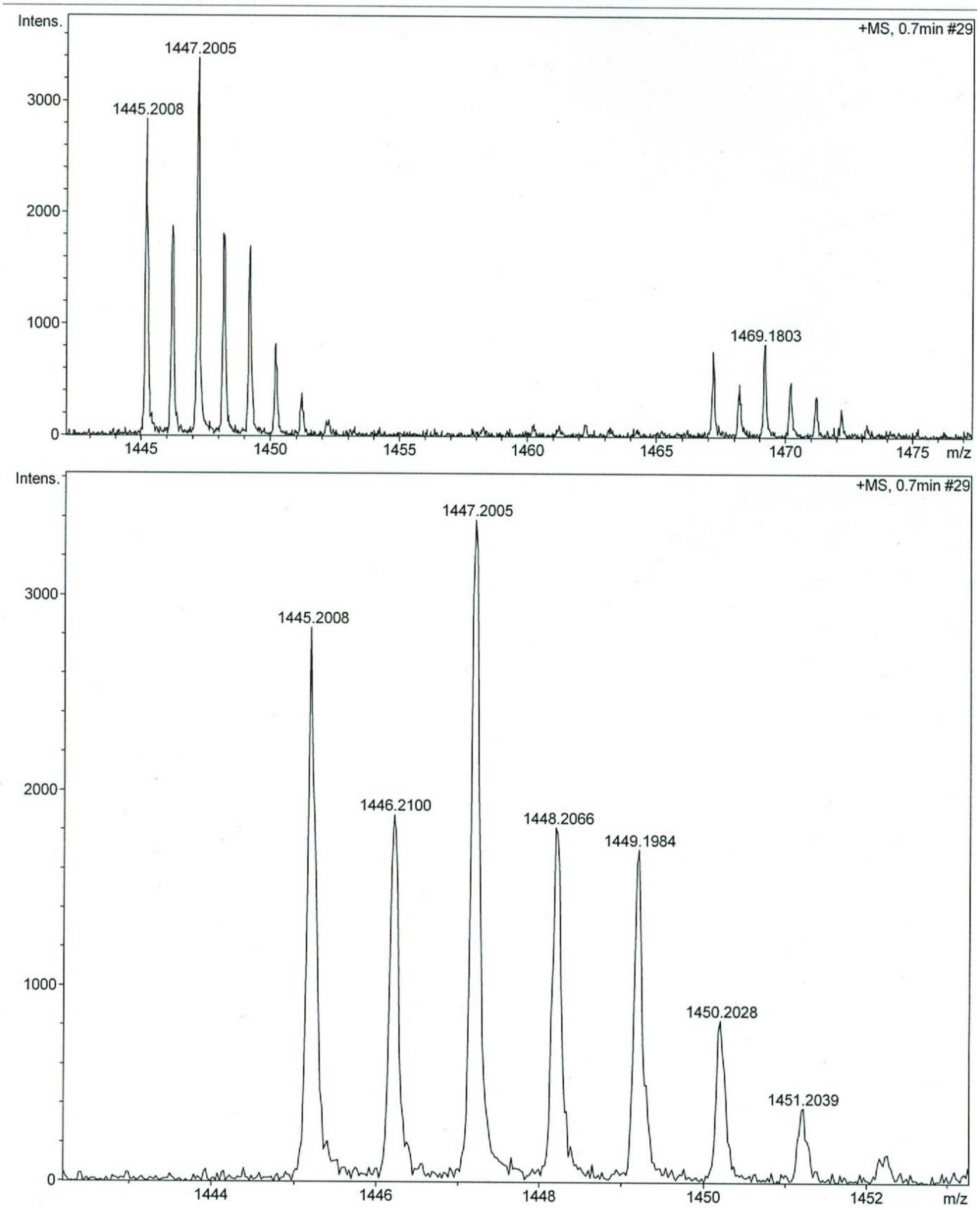

Figure S13: MS (ESI) spectrum of **10**.
